# Tumour stage and resection margin status are independent survival factors following partial pancreatoduodenectomy for duodenal adenocarcinoma

**DOI:** 10.1007/s00423-019-01779-w

**Published:** 2019-04-10

**Authors:** Kulbir Mann, T. Gilbert, S. Cicconi, R. Jackson, P. Whelan, F. Campbell, C. Halloran, J. Neoptolemos, P. Ghaneh

**Affiliations:** 1 Department of Molecular and Clinical Cancer Medicine, Institution of Translational Medicine, University of Liverpool, 2nd Floor Sherrington Building, Ashton Street, Liverpool, L69 3GE UK; 2 Statistics and Bioinformatics Unit, Cancer Research UK Liverpool Cancer Trials Unit, University of Liverpool, Block C, Waterhouse Building, 1-3 Brownlow Street, Liverpool, L69 3GL UK; 3 Department of Surgery, Royal Liverpool University Hospital, Prescot Street, Liverpool, L7 8XP UK; 4 Department of Pathology, Royal Liverpool University Hospital, Prescot Street, Liverpool, L7 8XP UK; 5 Department of Surgery, University of Heidelberg, Im Neuenheimer Feld 110, 69120 Heidelberg, Germany

**Keywords:** Duodenal cancer, Obstructive jaundice, Gastric outlet obstruction

## Abstract

**Introduction:**

There is limited published evidence on duodenal carcinoma due to its rarity. This study aimed to evaluate gastric outlet obstruction and obstructive jaundice along with pathological variables as survival factors in patients with duodenal adenocarcinoma following resection.

**Methods:**

Survival factor analysis was undertaken in patients undergoing duodenal cancer surgery from 1997 to 2015 in a single centre.

**Results:**

There were 57 patients of whom 18 had gastric outlet obstruction and 14 had obstructive jaundice. Fifty-three had a partial pancreatoduodenectomy and four had palliative bypass. Perioperative mortality and morbidity were 4% (2/53) and 47% (25/53) respectively in resected patients. With a median (95% confidence interval, CI) follow-up of 72 (57–86) months, median overall and recurrence-free survival was 38 months (95% CI 28–113) and 27 months (95% CI 18–83) respectively. The 1 and 3-year overall survival rates were 84% (95% CI 74–95) and 52% (95% CI 39–69) respectively. Median overall survival was 19 months in patients with gastric outlet obstruction vs 53 months in those without (*p* = 0.026) and 28 months in patients with obstructive jaundice vs 38 months in those without (*p* = 0.611). Univariate analysis revealed that tumour stage, resection margin status, pre-operative albumin status, gastric outlet obstruction and age were associated with poorer overall and recurrence-free survival but multivariate analysis confirmed only tumour stage and resection margin status to be significant.

**Conclusion:**

Whereas gastric outlet obstruction in duodenal cancer appeared to be an important survival factor following partial pancreatoduodenectomy, multivariate analysis showed that only tumour stage and resection margin status were the key independent survival factors. Further multicentre studies are required to elucidate further characteristics of duodenal carcinoma and develop neoadjuvant/adjuvant management strategies.

## Introduction

Duodenal adenocarcinoma is a rare malignancy; between 1998 and 2007, there were 2684 patients diagnosed in the UK and between 1997 and 2013, there were 2436 cases diagnoses in Germany [1]. The incidence is 0.4–0.6/10^5^ in men and marginally lower in women at 0.3–0.5/10^5^ with a 1-year survival of 35.9% and 16.1% at 5 years [2]. These survival figures differ markedly from those in Germany and the USA. The 1-year age-adjusted relative survival rates in Germany and the USA were reported as 67.2% and 68.8% respectively and the 5-year age-adjusted relative survival rates as 44.4% and 50.1% respectively [1]. Due to its low incidence, it is often grouped together with small bowel malignancy which accounts for 2% of all gastrointestinal tumours in the USA. Of all small bowel adenocarcinomas, 55.7% are located within the duodenum and 7% of periampullary tumours are duodenal in differentiation [3–5]. This infrequency in incidence has prevented the establishment of a thorough evidence base on which to derive uniform management and determine clinical and histopathological prognostic factors.

Initial clinical features are often insidious and more prominent when the lesion has grown to a size that can cause complications. There are vague symptoms of abdominal pain, nausea, vomiting, weight loss, fatigue and anorexia with some patients experiencing jaundice, gastrointestinal bleeding or gastric outlet obstruction [4, 5]. Investigations commonly involve direct visualisation and biopsy in the form of endoscopy or cross-sectional computed tomography imaging. Patients may have to undergo stenting via endoscopic retrograde cholangiopancreatography to provide relief of jaundice or require enteral or parenteral nutritional support. Surgical resection remains the mainstay of curative treatment, in the form of pancreatoduodenectomy or in rarer cases segmental duodenal resection [3]. Patients who have dysplastic duodenal polyps, either single or part of a hereditary condition such as familial adenomatous polyposis, may have undergone submucosal resection or a pancreas-preserving duodenectomy [6, 7]. There is very little published evidence regarding the use of neoadjuvant/adjuvant chemotherapy or radiotherapy. Some centres have treated duodenal adenocarcinoma similarly to periampullary tumours and use fluorouracil-based therapy and others as a colorectal malignancy and use oxaliplatin-based treatments [4, 5, 8, 9].

Due to the small number of cases, it has proved difficult to confidently identify survival factors although tumour size, lymph node status and resection margin status have often mentioned [5, 10–13]. In a single-centre study from Korea, the prognostic factors of 36 patients with duodenal cancer were analysed to find clear resection margin (R0), symptoms at initial admission, presence of lymph node metastasis, and perineural invasion significant only on univariate analysis [10]. The aim of this study was to include the specific presenting clinical features of obstructive jaundice and gastric outlet obstruction as part of the univariate and multivariate analysis of predictive survival factors.

## Material and methods

All patients undergoing surgical exploration with a view to partial pancreatoduodenectomy for presumed duodenal carcinoma from 1 January 1997 to 31 December 2015 at the Royal Liverpool University Hospital were reviewed from a prospectively maintained pancreatobiliary database. Hereditary dysplastic duodenal polyps, ampullary tumours of duodenal origin and neuroendocrine lesions were excluded from the study. Data were obtained retrospectively from patient case notes and electronic records.

**Table 1:** Patient cohort characteristics

	Total	Gastric outlet obstruction			Obstructive jaundice		
	*N* = 57	*N* = 51			*N* = 52		
		No	Yes	*p* value	No	Yes	*p* value
		*n* = 33	*n* = 18		*n* = 38	*n* = 14	
Gender							
Female	32 (56%)	13 (39%)	10 (56%)	0.378	16 (42%)	7 (50%)	0.755
Male	25 (44%)	20 (61%)	8 (44%)		22 (58%)	7 (50%)	
Age (years)							
Median	71	71	69	0.252	710	74	0.129
IQR	(63.0–74.0)	(66.0–74.0)	(60.0–72.8)		(63.2–73.0)	(68.2–75.5)	
Time to operation (days)							
Median	41	47	32	0.001	40	48	0.323
IQR	(32.0–54.5)	(36.0–58.0)	(25.0–41.0)		(30.5–48.8)	(35.0–57.0)	
No. of observations available	47	29	17		34	13	
Length of stay (days)							
Median	26	18	40	0.001	23	32	0.146
IQR	(15.5–40.5)	(13.5–31.0)	(31.2–47.5)		(14.0–39.0)	(21.2–49.0)	
No. of observations available	47	31	16		33	14

**Table 2:** TNM staging for patients undergone successful resection

	Total	Gastric outlet obstruction			Obstructive jaundice		
	*N* = 53	*N* = 47			*N* = 48		
		No	Yes	*p* value	No	Yes	*p* value
		*n* = 31	*n* = 16		*n* = 37	*n* = 11	
T stage							
1	2 (4%)	2 (6%)	0 (0%)	0.068	1 (3%)	1 (9%)	0.569
2	7 (13%)	7 (23%)	0 (0%)		5 (14%)	2 (18%)	
3	15 (28%)	6 (19%)	7 (44%)		12 (32%)	2 (18%)	
4	29 (55%)	16 (52%)	9 (56%)		19 (51%)	6 (55%)	
N stage							
0	17 (32%)	10 (32%)	6 (38%)	0.794	12 (32%)	4 (36%)	0.217
1	21 (40%)	13 (42%)	5 (31%)		12 (32%)	6 (55%)	
2	15 (28%)	8 (26%)	5 (31%)		13 (35%)	1 (9%)	
R status							
0	43 (81%)	28 (90%)	9 (56%)	0.020	28 (76%)	10 (91%)	0.416
1	10 (19%)	3 (10%)	7 (44%)		9 (24%)	1 (9%)	

Data retrieved included patient demographics, timing of management, peri/post-operative details, tumour histology and stage, length of stay, adjuvant treatment, re-interventions/readmissions and survival data. The TNM staging was standardised to the latest UICC version 8 [14]. Time to operation parameters are from initial diagnostic investigation to date of procedure and includes the time to multidisciplinary meeting. Length of stay results are overall time in the hospital inclusive of early pre-operative admissions. Post-operative complications were reported according to the Clavien-Dindo classification [15]. Patients who had a raised bilirubin of greater than 40 μmol/L were considered to have obstructive jaundice, and a record was made of pre-operative endoscopic retrograde cholangiopancreatography (ERCP) and percutaneous transhepatic cholangiography (PTC). Those patients admitted or transferred to this institution with symptoms of persistent vomiting and being unable to tolerate diet, correlating with an obstructive diagnosis on gastroscopy or oral contrast computer tomography (CT) were recorded as gastric outlet obstruction.

Continuous variables are presented with their median and interquartile range (IQR), while categorical variables are described as frequency counts and proportion percentages. The Fisher exact test or Wilcoxon-Mann-Whitney test were performed as appropriate in order to compare proportions and distributions. Survival analysis was carried out for time to death and time to recurrence. Time to death was defined as the difference between date of death (or latest follow-up visit for living patients) and date of diagnosis. Time to recurrence was defined as the difference between the date of imaging demonstrating recurrence or date of death (or latest follow-up visit for living patients free from recurrence) and date of operation. For both these responses, median survival estimates from Kaplan-Meier method and their 95% confidence intervals (CIs) are reported. Cox proportional hazard models were initially fitted for each covariate independently and are presented as hazard ratios (HR) with 95% CI [16]. Prognostic factors with a *p* value less than 0.05 were further explored in multivariable settings using backward selection technique based on Akaike Information Criterion for selecting the best model [17]. Proportional hazard assumption for Cox models was assessed with Schoenfeld residuals [18]. Kaplan-Meier curves and log-rank test were applied to investigate the difference in survival distribution between the clinical subgroup of interest. Median follow-up time was estimated with the reverse Kaplan-Meier method [19]. A *p* value less than 0.05 was considered to be significant. All statistical analyses were performed with R version 3.3.0.

## Results

**Fig. 1:**
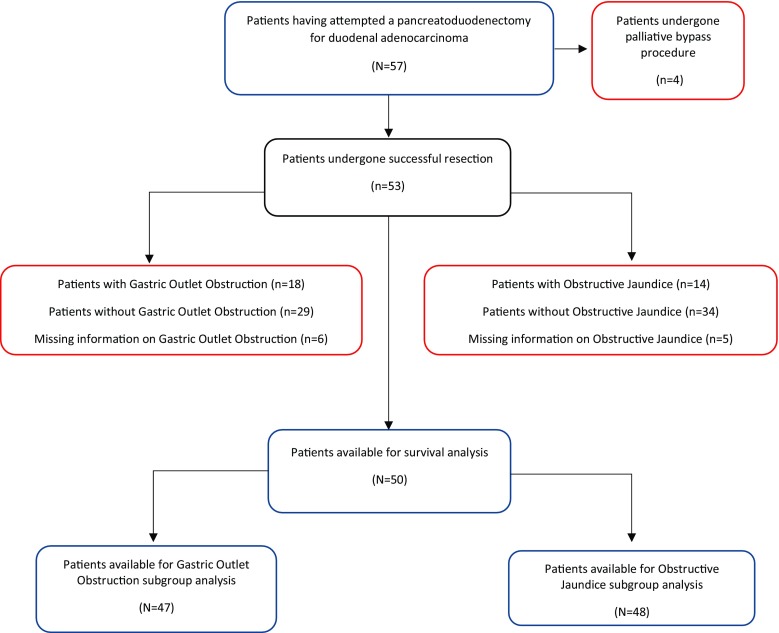
A CONSORT flow diagram demonstrating the cohorts of patients with obstructive jaundice and gastric outlet obstruction

There were 57 patients who had an attempted partial pancreatoduodenectomy for duodenal adenocarcinoma during the 18-year study period. The median age was of 71 (IQR 63.0–74.0) years and the female to male ratio of 1:0.78. Median time from the diagnosis to date of operation was 41 (IQR 32.0–54.5) days. Eighteen (32%) patients presented with gastric outlet obstruction, and 12 of them were admitted prior to operation for enteral or parenteral nutritional support. There were significantly lower levels of pre-operative serum albumin in patients presenting with gastric outlet obstruction compared to those without 34.0 (IQR 34.0–42.0) vs 38.0 (IGR 27.5–37.8) g/L, respectively, *p* = 0.014. Fourteen patients (25%) presented at this or local institutions with obstructive jaundice and eight of them had successful pre-operative ERCP and stenting. Five patients had successful PTC and stenting, with a single failure. Despite these interventional procedures, there were still six patients who had bilirubin levels > 100 μM/L on the day of operation. Information was not recorded in six patients with gastric outlet obstruction and five with obstructive jaundice respectively (Fig. 1). There were four patients who had both gastric outlet obstruction and obstructive jaundice but, for the purpose of this analysis, these were treated separately. Median time to operation was significantly shorter in patients with gastric outlet obstruction than without, 32 (IQR 25.0–41.0) days vs 47 (IQR 36.0–58.0) days respectively [*U* = 390, *p* value = 0.001] (Table 1).

Fifty-three (93%) patients underwent a curative resection, and four (7%) had a palliative gastric/biliary bypass procedure for local invasion or metastatic spread. Fifteen (28%) patients undergoing resection had a classic Kausch Whipple procedure, and 38 (72%) had a pylorus preserving pancreatoduodenectomy with three patients requiring a right hemicolectomy to complete tumour resection. A single patient had a splenectomy because of a suspicious lesion on pre-operative staging, which was actually a lymphangioma. All histopathology results were collated including confirmation biopsies for patients in the bypass group. Fifty resected specimens were intestinal type duodenal carcinoma; two were mucinous type cancer and one with signet cell carcinoma. Nine patients (17%) had tumour (T) staging of 1 or 2, 36 patients (68%) had lymph node (LN) involvement and 43 patients (81%) had tumour free resection margins (R0). It is worth noting that patients who had a right hemicolectomy were not more prevalent within a gastric outlet obstruction or obstructive jaundice group (Table 2).

**Fig. 2:**
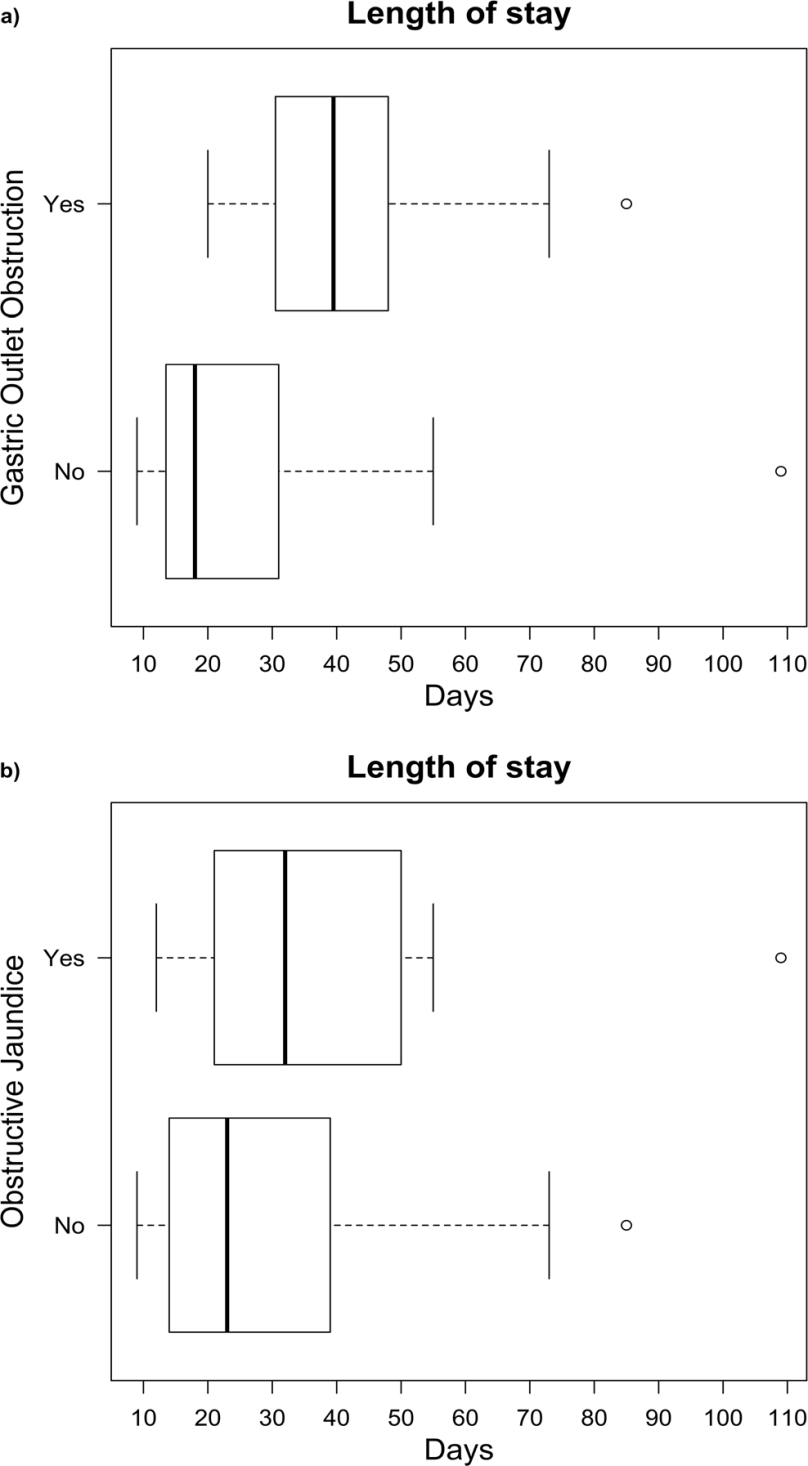
Box plot distributions of length of stay in patients with **a** gastric outlet obstruction and **b** obstructive jaundice preoperatively

There were two (4%) post-operative deaths following resection both due to myocardial infarction. There were 30 post-operative complications in 24 (47%) patients: 21 Clavien-Dindo grade II, six with grade III and three with grade IV. Fourteen (26%) patients had intra-abdominal collections, of which six (11%) were associated with pancreatic leaks and they were managed conservatively with antibiotics in eight cases and interventional drainage in six cases. There were three incidences of delayed gastric emptying (6%) that required total parenteral nutrition. The remaining post-operative morbidities consisted of six (11%) patients with surgical site infections, five (9%) patients with respiratory complications, one (2%) patient with an acute kidney injury and one (2%) patient with an upper arm deep vein thrombosis. No significant differences in post-operative course or complications rates were found in patients presenting with gastric outlet obstruction or obstructive jaundice compared to those without either. The median length of total hospital stay (which included pre-operative admission for nutritional supplementation) in patients with gastric outlet obstruction was 40 (IQR 31.2–47.5) days compared to 18 (IQR 13.5–31.0) days for patients without gastric outlet obstruction [*U* = 102, *p* value = 0.001] (Fig. 2). The median pre-surgery length of hospital stay in patients with gastric outlet obstruction was 13 (IQR 10.1–26.0) days compared to 0 (IQR 0.4–5.9) days in those without gastric outlet obstruction.

Eighteen (32%) patients underwent adjuvant chemotherapy, and two (4%) patients had adjuvant chemoradiotherapy. Ten were treated with capecitabine as a single therapy regimen, whereas three patients had gemcitabine monotherapy and three patients had 5-fluorouracil monotherapy. A further four patients had dual drug regimens involving folinic acid, 5-fluorouracil, capecitabine and oxaliplatin. Thirty (53%) patients that did not receive any adjuvant chemotherapy because of poor performance status in 16 patients, oncology advice in nine patients, personal choice with two patients and death in three patients. Records were not available in seven cases. Though a lack of consistent therapies, the use of any adjuvant therapy did not have a significant effect on patient survival, [HR = 0.88 (95% CI 0.40–1.92), *p* value = 0.742].

**Fig. 3:**
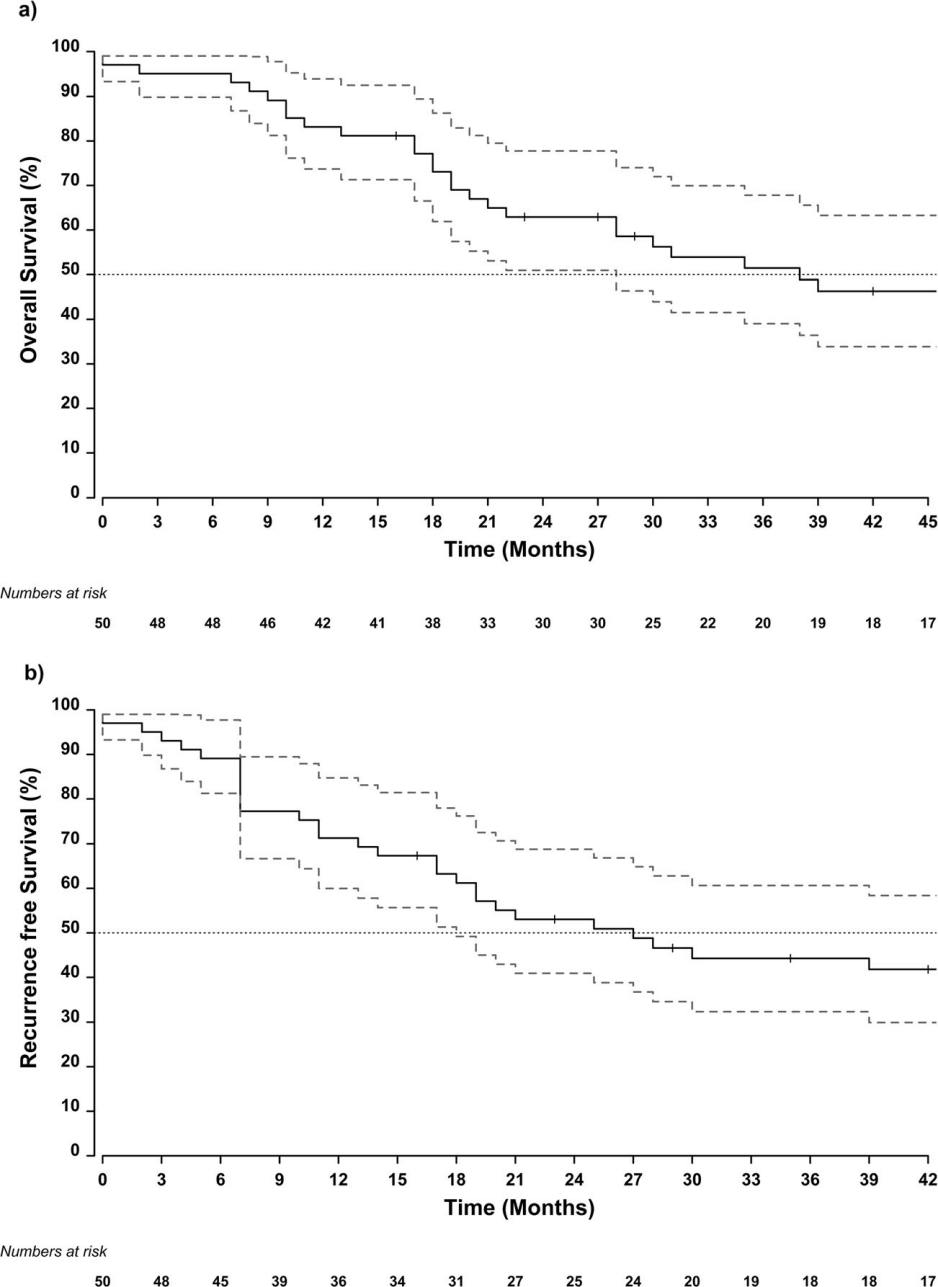
Kaplan-Meier estimates of median survival and recurrence-free survival with 95% confidence intervals of duodenal adenocarcinoma patients with **a** gastric outlet obstruction and **b** obstructive jaundice preoperatively

**Fig. 4:**
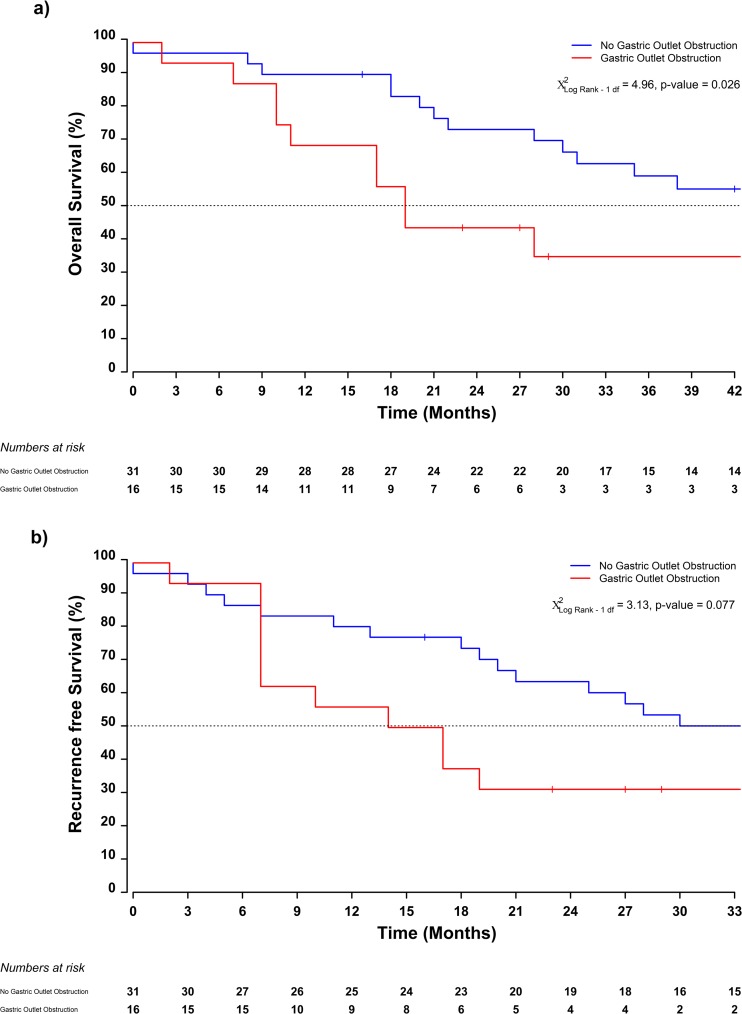
Kaplan-Meier estimates of median survival by clinical sub groups of **a** obstructive jaundice and **b** gastric outlet obstruction

**Fig. 5:**
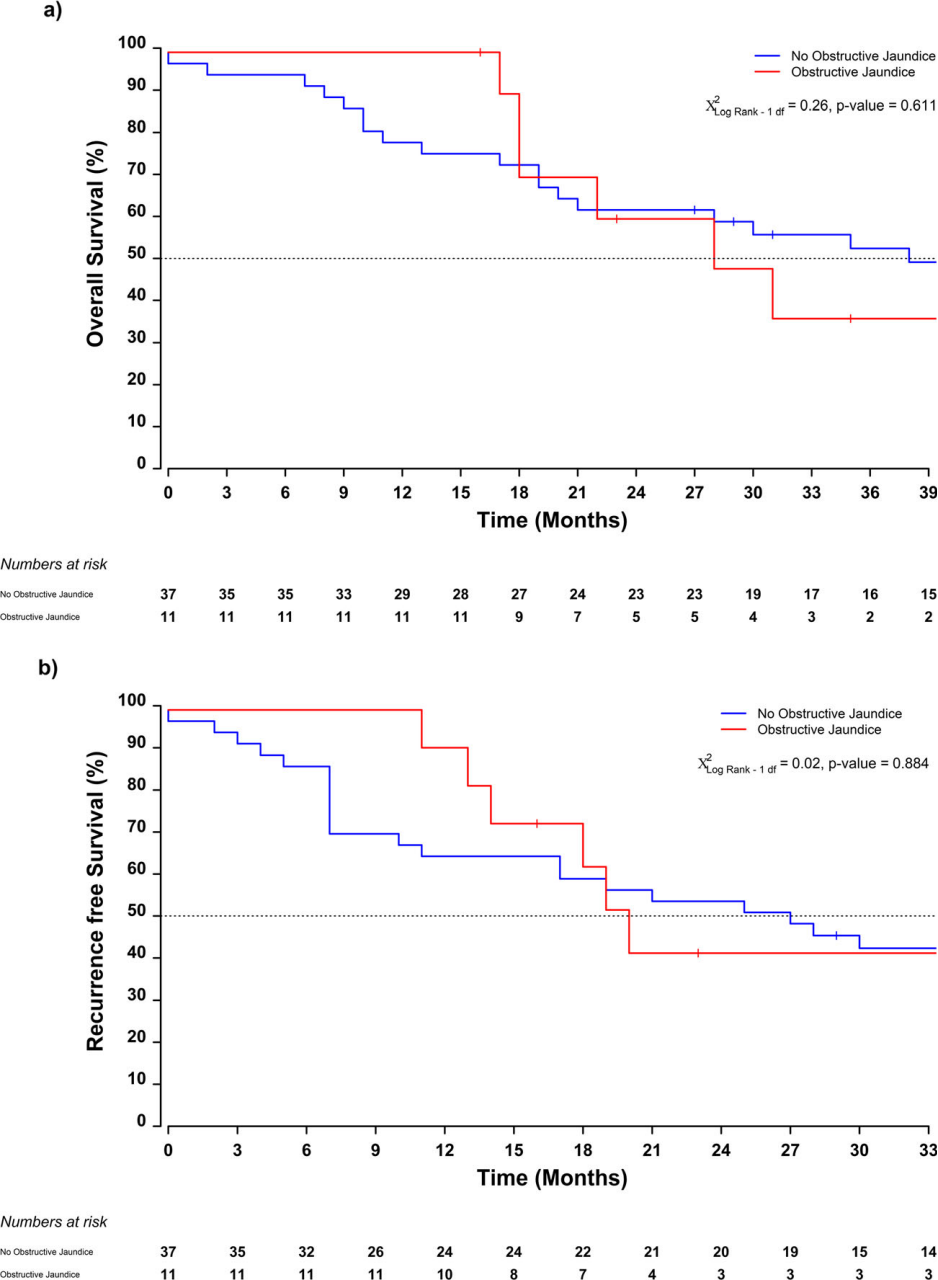
Kaplan-Meier estimates of median time to recurrence by clinical sub groups of **a** obstructive jaundice and **b** gastric outlet obstruction

The median follow-up time was 72 (95% CI 57–86) months. The median overall survival (OS) time was 38 (95% CI 28–113) months, with 1- and 3-year survival rates of 84% (95% CI 74–95) and 53% (95% CI 39–69) respectively. The median recurrence-free survival (RFS) was 27 (95% CI 18–83) months, with 1- and 3-year recurrence-free survival rates of 72% (95% CI: 61–86) and 45% (95% CI: 33–61) respectively (Fig. 3). The median OS time was 19 months (95% CI 11–unobtainable) in the group of patients presenting with gastric outlet obstruction and 53 (95% CI 31–unobtainable) months in patients presenting without [log-rank *χ*^2^_df = 1_ = 4.96, *p* value = 0.026] (Fig. 4a). Although without reaching statistical significance, similar trends were reported with respect to time to recurrence, 16 (95% CI 7–unobtainable) months vs 47 (95% CI 21–unobtainable) months for patients with and without gastric outlet obstruction respectively [log-rank *χ*^2^_df = 1_ = 3.13, *p* value = 0.077] (Fig. 4b). No differences in overall survival or recurrence-free survival were detected between patients having obstructive jaundice or not (Fig. 5).

Univariate Cox models showed that resection margin status, tumour stage and pre-operative albumin levels were related to both time to death and time to recurrence (Tables 3 and 4). Gastric outlet obstruction was also associated with overall survival, and age was associated with recurrence-free survival. Multivariable Cox regression identified resection margin status [HR = 5.83 (95% CI 2.25–15.07), *p* value < 0.001] and tumour stage [HR = 2.27 (95% CI 1.02–5.04), *p* value = 0.040] as predictive factors for overall survival (Table 5). The best model for recurrence-free survival included resection margin status [HR = 4.48 (95% CI 1.95–10.28), *p* value < 0.001], age [HR = 1.06 (95% CI 1.01–1.11), *p* value = 0.012] and tumour stage [HR = 2.28 (95% CI 1.07–4.86), *p* value = 0.070], although the latter did not reach the significance level in the multivariable setting (Tables 5 and 6).

**Table 3:** Univariate Cox models for time to death

Variable	Hazard ratio (95% CI)	Log-rank *χ*^2^	*p* value
Gender			
Female	1	0.15	0.697
Male	1.15 (0.56–2.36)		
Age (years)	1.03 (0.99–1.08)	2.03	0.154
T stage			
3−	1	8.59	0.003
4	2.97 (1.39–6.35)		
N stage			
0	1	1.92	0.166
1+	1.76 (0.78–3.95)		
R status			
0	1	25.74	< 0.001
1	7.88 (3.11–19.96)		
Albumin (g/L)	0.94 (0.89, 0.99)	5.33	0.021
Gastric outlet obstruction			
No	1	4.96	0.026
Yes	2.30 (1.08–4.90)		
Obstructive jaundice			
No	1	0.26	0.611
Yes	1.25 (0.53–2.96)		
Adjuvant treatment			
No	1	0.11	0.742
Yes	0.88 (0.40–1.92)

**Table 4:** Univariate Cox models for time to recurrence

Variable	Hazard ratio (95% CI)	Log-rank *χ*^2^	*p* value
Gender			
Female	1	0.10	0.758
Male	1.12 (0.56–2.23)		
Age (years)	1.05 (1.00–1.09)	3.59	0.058
T stage			
3−	1	6.82	0.009
4	2.56 (1.23, 5.31)		
N stage			
0	1	2.34	0.126
1+	1.85 (0.83, 4.12)		
R status			
0	1	18.34	< 0.001
1	5.06 (2.23, 11.49)		
Albumin (g/L)	0.95	4.57	0.032
	(0.90, 1.00)		
Gastric outlet obstruction			
No	1	3.13	0.077
Yes	1.93 (0.92–4.03)		
Obstructive jaundice			
No	1	0.02	0.884
Yes	1.07 (0.45, 2.51)		
Adjuvant treatment			
No	1	0.00	0.975
Yes	0.99 (0.47–2.082)

**Table 5:** Multivariate Cox model for time to death

Variable	Hazard ratio (95% CI)	Log-rank χ^2^	*p* value
R status			
0	1	16.20	< 0.001
1	5.83 (2.25–15.07)		
T stage			
3-	1	4.22	0.040
4	2.27 (1.02–5.04)

**Table 6:** Multivariate Cox model for time to recurrence

Variable	Hazard ratio (95% CI)	Log-rank *χ*^2^	*p* value
R status			
0	1	12.68	< 0.001
1	4.48 (1.95–10.28)		
T stage			
3−	1	3.29	0.070
4	2.28 (1.07–4.86)		
Age (years)	1.06 (1.01–1.11)	6.29	0.012

## Discussion

This study has shown that advanced tumour stage, positive resection margins, low pre-operative albumin levels, the presence of gastric outlet obstruction and advanced age were each associated with shorter overall and recurrence-free survival following partial pancreatoduodenectomy for duodenal cancer. On multivariate analysis however, only tumour stage and resection margin status were of independent significance. This indicates that whereas gastric outlet obstruction in duodenal cancer appeared to be an important survival factor per se, the symptom of obstruction signified a larger tumour diameter. The challenges in removing a larger tumour are reflected in the greater frequency of positive margins in this group and points to the need for an effective neoadjuvant and/or adjuvant strategy.

The Royal Liverpool University Hospital serves a catchment area of over 400,000 people and acts as a tertiary referral centre for hepatobiliary disease for a much larger populace of approximately 2.4 million and represents a relatively large single-centre experience. This compares to a series of 178 patients over 13 years from six other large centres in the UK [13], whilst other studies including those from the USA and Japan have reviewed from 21 to 122 patients [5, 10, 12, 20–24]. In the present study, only four (7%) patients underwent a palliative procedure which is less than that observed in two larger cohort studies reflecting the quality of pre-operative workup and technical surgery [12, 13, 25]. There were only two (4%) post-operative deaths both due to myocardial infarction. The morbidity rate of 47% is similar to those published in previous studies of approximately 40–46% [5, 12, 13, 22, 23]. The majority (70%) of complications were Clavien-Dindo grade II requiring only medical intervention. The proportion of patients with a pancreatic leak was 11%, in keeping with a published range of 9.3–28.9% [5, 12, 13, 21, 22]. The delayed gastric emptying rate of 6% is lower than the reported rates of 12.5–24% [5, 12, 13, 22, 23].

The median overall survival in duodenal adenocarcinoma is approximately 35.5–83 months in published series [5, 12, 13, 22, 23]. The median overall survival of 38 months in our study is at the lower end of this range but much improved from previous United Kingdom figures and recent German and US publications [1, 2]. Negative resection margin status and tumour stage III or lower were associated with longer survival. Published series have not consistently found lymph node status to be a significant prognostic factor [4, 5, 12, 13, 25, 26]. Interestingly, age seemed to be a prognostic factor for recurrence-free survival which has been described recently in a German observational study [27]. A study of small bowel cancers based on the USA Surveillance, Epidemiology, and End Results (SEER) database from 2004 to 2013 found that duodenal localization was associated with worse overall survival and that young age, retrieval of more than 12 regional lymph nodes, less advanced stage and married matrimonial status were positive, independent prognostic factors [1].

There is no standard of adjuvant chemotherapy in patients with resected duodenal adenocarcinoma. In our study, 20 patients (35%) underwent chemotherapy or chemoradiotherapy, and ten of these had the same drug regimen (capecitabine). The ESPAC-3 periampullary trial, which included 80 intestinal type tumours, found an increase in median survival of 7.9 months comparing a chemotherapy group (5-fluorouracil plus folinic acid) to an observation group [28]. This is the only randomised phase three trial pertinent to duodenal carcinoma, but there have been three phase II studies that have observed the benefit of chemotherapy on advanced small bowel tumours. Response rates of 18.4% were reported using 5-fluorouracil, doxorubicin, and mitomycin C and a rate of 50% more recently utilising capecitabine and oxaliplatin [8, 9]. Adding irinotecan to capecitabine and oxaliplatin has given a response rate of 37% in the latest phase II trial of 33 patients [29]. Due to the lack of robust adjuvant chemotherapy trials the rate of adjuvant therapy is quite variable [4, 5, 12, 13]. The use of radiotherapy in duodenal cancer has been studied even less, and propensity score-matched analysis of national observational oncological data from the US and South Korea has been reported. They observed that radiation provides minimal survival benefit in addition to adjuvant chemotherapy but may have a role in locally advanced disease [30, 31].

There have been no published studies specifically reviewing the effects of obstructive jaundice and gastric outlet obstruction on the outcomes of duodenal adenocarcinoma. The Korean single-centre study grouped together presenting symptoms (abdominal pain, vomiting, gastrointestinal bleeding, dyspepsia, weight loss, jaundice, diarrhoea and oedema) as part of a multivariate analysis and found no significant prognostic factors [10]. In the present study, we included the specific presenting clinical features of obstructive jaundice and gastric outlet obstruction as part of the predictive modelling, given that one or other of these clinical features might influence clinical decision-making. The systemic effects of obstructive jaundice on patients with pancreatic ductal adenocarcinoma have been well established, and preoperative biliary drainage with metal stenting is routine in some centres. However, others undertake immediate resection without biliary stenting and some centres are employing a “fast track” pathway for pancreatoduodenectomy procedures [32–35]. In the present study, obstructive jaundice at presentation was not associated with pathological staging, resectability, post-operative complications and survival. Patients presenting with gastric outlet obstruction required a longer overall length of hospital stay to improve nutritional status. Pre-operative serum albumin levels were significantly lower in these patients with gastric outlet obstruction reflecting the nutritional deficit and the development of a cachexia associated with cancer [36]. Although gastric outlet obstruction was associated with reduced survival on univariate analysis, this was not supported once other prognostic factors were taken onto account. The impact of gastric outlet obstruction is complex because it incorporates greater proportions of high tumour staging and positive resection margins which are independently significant factors. As a presenting symptom, it reflects multiple aspects of tumour staging and the study may be underpowered to demonstrate its significance. Its importance should not be overlooked and efficient pre-optimisation and meticulous operative preparations should be undertaken.

The limitations of this study are the relatively small number of patients and its retrospective nature. The study is strengthened however in being from a single centre and with sufficient number of events for each of the outcomes for statistical modelling. The duration of our study is over 18 years and there have been significant changes to the clinical practice and post-operative management of patients, especially with the implementation of Enhanced Recovery after Surgery (ERAS) protocols. This has had an impact on the post-operative outcomes and length of stay of patients [4, 5, 12, 13].

## Conclusion

Partial pancreatoduodenectomy with sufficient lymph node clearance is the only effective treatment for duodenal cancer achieving longer disease free and overall survival. From a practice perspective, patients presenting with obstructive jaundice and/or gastric outlet obstruction should not be assumed to have a poor prognosis but be considered for curative resection with an emphasis on nutrition and a meticulous operative approach. Tumour stage and resection margin status are key independent survival factors indicating a need for effective neoadjuvant and/or adjuvant treatments.
